# Impact of moderate-to-severe psoriasis on quality of life in China: a qualitative study

**DOI:** 10.1186/s12955-021-01902-w

**Published:** 2021-12-24

**Authors:** Hua Zhong, Huan Yang, Zhuxin Mao, Xiaoyun Chai, Shunping Li

**Affiliations:** 1 Department of Dermatology, Qilu Hospital, Shandong University, Jinan, 250012 China; 2 Centre for Health Management and Policy Research, School of Public Health, Cheeloo College of Medicine, Shandong University, Jinan, 250012 China; 3 NHC Key Laboratory of Health Economics and Policy Research (Shandong University), Jinan, 250012 China; 4 Center for Health Preference Research, Shandong University, Jinan, 250012 China; 5 School of Insurance, Southwestern University of Finance and Economics, Chengdu, 611130 China

**Keywords:** Psoriasis, Quality of life, Qualitative study, China

## Abstract

**Purpose:**

Psoriasis is a serious health problem. Since limited research has investigated the impact of psoriasis on the quality of life of patients with moderate-to-severe psoriasis, this study aimed to explore this issue.

**Methods:**

A qualitative study was conducted with 22 psoriasis patients from two cities in Shandong province of eastern China participating in one-to-one semi-structured in-depth interviews.

**Results:**

Thematic analysis generated five major themes: (1) Symptoms, symptoms management and pain; (2) Functioning and activities of daily living (ADLs); (3) Psychological impact; (4) Social impact; (5) Employment and finances.

**Conclusion:**

Our study detailed the effects of psoriasis on patients’ symptoms, symptoms management and pain, functioning and activities of daily living (ADLs), psychological impact, social impact, employment and finances. These data can provide a reference for studying the quality of life in patients with psoriasis.

## Introduction

Psoriasis is a chronic and incurable inflammatory skin disease that is characterized by epidermis hyperproliferation and can cause thick, red, scaly lesions anywhere on the body [1]. The reported prevalence of psoriasis ranges between 0.09% [2] and 11.4% [3], making psoriasis a common health problem. In China, the prevalence of psoriasis is 0.47% [4], showing an upward trend from 0.12% [5]. Psoriasis has equal gender prevalence [6] and can occur at any age. It has been reported to appear at birth and in the elder population but is most common in the age between 50 and 69 [7].

The aetiology of Psoriasis is multifactorial [8], incorporating biological, environmental, and psychosocial factors. The disease can be regarded as a psychosomatic disorder [9, 10]. Factors such as genetic heritage, physiological and psychogenic stress, smoking, and hormonal changes can play a role in the exacerbation of the disease [6]. Psoriasis is associated with comorbidities including metabolic syndrome, type 2 diabetes mellitus, depression, and non-alcoholic fatty liver disease [11, 12]. It is also associated with a higher rate of mortality due to an increased risk of cardiovascular disease, myocardial infarction and stroke [13].

Although psoriasis generally does not affect survival, it has a significant detriment to quality of life (QoL) [14]. Research has shown that patients with psoriasis report physical discomfort and limitations in their daily activities and social contacts [15, 16]. They also experience severe itching and pain, as well as plaques coming loose and falling from their skin [17]. These symptoms can cause significant distress and feelings of embarrassment and shame, with significant impacts on functioning across social and employment domains [17]. The visible nature of psoriasis can cause stigma, lowered self-image, depression, anxiety, and suicidal ideation [18]. Particularly, if a patient has psoriasis on the head, they were highly possible to have anxiety and depression [19–21]. Even in mild cases, psychological impairment has also been observed [22], and social avoidance behavior may occur. For example, patients will refuse to participate in sports requiring bare skin, such as swimming, playing football, etc. [23, 24]. These avoidance behaviors may make it difficult for patients to make friends and obtain emotional support and comfort.

Over the past decade, scholars have used various quantitative methods to evaluate QoL among patients with psoriasis [25], the perceptions of quality of life can differ among people from different ethnic groups and cultural backgrounds [25, 26]. In China, limited studies have used qualitative approaches to attain an in-depth understanding of the impact of moderate-to-severe psoriasis on the quality of life of those affected, therefore, we conducted a qualitative study to understand the QoL of Chinese patients with psoriasis, which may enable dermatologists to provide better comprehensive medical care to patients. Having reviewed relevant literature, in this research, we made hypotheses that psoriasis can affect a patient's QoL physically (symptoms, pain and functioning), mentally (psychological impacts) and socially (social and employment aspects).

## Methods

### Study setting and design

A qualitative methodology was used to ensure as much openness for novel and unexpected findings as possible while allowing a detailed description of the various facets of the research topic [27]. This study was carried out at two dermatological inpatient clinics in Jinan and Qingdao, Shandong Province. Patients were recruited consecutively at the clinics. The recruitment period was from November 10, 2018, to March 16, 2019. Demographic data and clinical characteristics, including disease severity, duration of psoriasis and QoL data, were collected.

This study was approved by the Ethics Committee of Qilu Hospital, Shandong University (KYLL-2018-361).

### Participants

A total number of 22 (12 from Qilu Hospital of Shandong University, 10 from Qingdao Municipal Hospital) patients with psoriasis were recruited to participate in interviews. The inclusion criteria were age 18 years or older and a diagnosis of moderate-to-severe psoriasis. Patients with psoriatic arthritis and those participating in other clinical trials were excluded. The sociodemographic and clinical characteristics of the 22 participants are presented in Table 1. There were 14 men (63.6%) and 8 women (36.4%), with a mean age of 42.6 ± 12.9 years (range 18–70 years), and most of them (68.2%) were married. In the level of education, one half (50.0%) had completed high school or technical secondary school. With regards to occupation, 16 participants (72.7%) had jobs. The most frequent clinical type was plaque psoriasis. The mean duration of psoriasis was 12.5 ± 10.5 years (range 1–41 years). The majority of the participants (59.1%) were in moderate severity symptoms.

**Table 1:** Demographic and clinical characteristics of participants

Characteristics	N (%) or mean ± SD (n = 22)
*Gender*	
Male	14 (63.6)
Female	8 (36.4)
*Age (years)*	
< 35	7 (31.8)
35–50	9 (40.9)
> 50	6 (27.3)
*Educational level*	
No school or primary school	2 (9.1)
Secondary school	2 (9.1)
High school or technical secondary school	11 (50.0)
University degree and above	7 (31.8)
*Marital status*	
Married	15 (68.2)
Divorced or widowed	1 (4.5)
Single	6 (27.3)
*Occupation*	
Public institutions	4 (18.2)
Company employee	6 (27.3)
Freelancers	4 (18.2)
Peasants	2 (9.1)
Students	1 (4.5)
Unemployment	5 (22.7)
*Psoriasis area and severity index (PASI)*	
7–12 (moderate)	13 (59.1)
> 12 (severe)	9 (40.9)
*Duration of psoriasis (years)*	
Mean ± SD	12.5 ± 10.5
Range	1–41

### Data collection

The interviewers had extensive knowledge of psoriasis and received training to conduct qualitative research. Trained interviewers worked in collaboration with dermatologists. The meaning of collaboration is that dermatologists would explain the study to patients before formal interviews, so that patients can understand the interview process and their trust in interviewers can be enhanced. Two patients who were eligible for the interview refused to participate, because they were not willing to take time in participating the interview. The overall participation rate as a proportion of all eligible participants is 91.7%. Patients who gave verbal informed consent were recruited, followed by formal interviews with interviewers. One-to-one interviews were conducted in a quiet room at the clinics with special precautions for privacy.

Each interview consisted of three sections. In Sect. 1, patients were asked about their socio-demographic characteristics including gender, age, marital status, education levels and employment status. Section 2 was about the clinical characteristics of patients, such as disease severity and duration of disease. The severity of psoriasis was quantitatively assessed by a dermatologist using the Psoriasis Area and Severity Index (PASI) [28]. PASI results in a score ranging from 0 to 72, and it is usually re-grouped into three themes implying three severity levels of psoriasis: PASI < 7 (mild severity), PASI 7–12 (moderate severity), and PASI > 12 (severe severity) [28]. Section 3 focused on the impact of psoriasis on QoL.

In our study, we chose to use one-to-one interviews instead of a focus group. This was because, on the one hand, each interviewer may have different opinions and experiences with psoriasis, one-to-one interviews allowed for an in-depth discussion with each interviewee; on the other hand, a one-to-one interview can better protect the privacy of our interviewees, since the interviewees may consider some questions were sensitive and may be reluctant to talk in front of too many people. In our interview, the patients first needed to answer some open questions, such as what happened to their life after suffering from psoriasis. Then according to the patients’ answers, interviewers can have follow-up questions and in-depth discussions to explore issues that emerged during interviews. Table 2 presents our main interviewing questions and potential follow-up questions.

**Table 2:** Interview manual

Main questions
How do you feel today? How is your life today?
What kind of treatment do you receive for treating psoriasis?
How do you like your treatment?
How is psoriasis affecting your life? Can you give me some examples?
Could you describe your daily life after you were diagnosed with psoriasis?
Potential follow-up questions
What kind of physical or physiological discomfort does it bring to you from psoriasis?
What are the psychological or spiritual effects of suffering from psoriasis?
What is the impact of getting psoriasis on your family or social relationships?
What other aspects do you feel have a great impact on your health during the treatment or treatment of psoriasis?

The average length of the interviews was approximately 40 min. We conducted data collection and data analysis simultaneously. When we conducted 19 interviews, we found that no new concepts emerged. We continued to recruit 3 more people and confirmed that new interviews did not provide us with new content, indicating that saturation was reached, then we stopped our data collection. All interviews were digitally recorded. Confidentiality was ensured by assigning numbers to each patient rather than using their names. Each patient was given a vacuum beverage bottle to compensate for their time.

### Data analysis

All interviews were transcribed verbatim. Thematic analysis was conducted to analyze the data [29]. The transcripts were read several times by two researchers independently of each other to achieve an overall conception, after which they developed a coding list through consensus discussion with a third researcher. The two researchers then coded the transcripts line by line according to the coding list. Codes were combined or contrasted, then grouped into categories to generate themes. The two researchers finalized the themes and sub-themes through consensus discussion with a third researcher, the interrater reliability of the coding was established with a third coder. This triangulation was done to improve the credibility, consistency and reflexivity of the research. Codes were then grouped to generate themes. All analyses were performed in Chinese and the final results were translated and reported into English. As a result of this process, definitions and names for codes were generated which were collated into four key themes. The analyses were conducted in Microsoft Excel.

## Results

### Key theme

Five themes emerged in this study, including “symptoms, symptoms management and pain”, “functioning and activities of daily living (ADLs)”, “psychological impact”, “social impact”, “employment and finances”, Table 3 shows the detailed category system and subcategories.

**Table 3:** Frequency of category and subcategory

Category	Frequencies of categories N (%)	Subcategory	Frequencies of subcategories N (%)
Symptoms, symptoms management and pain	21 (95.5%)	Hurting	9 (20.0)
		Itching	8 (17.8)
		Bleeding	7 (15.6)
		Burning/stinging	6 (13.3)
		Irritated	5 (11.1)
		Flaking	5 (11.1)
		Discomfort	5 (11.1)
Functioning and activities of daily living (ADLs)	20 (90.9%)	Summer activities	10 (18.9)
		Choose of clothes	8 (15.1)
		Good sleep	8 (15.1)
		Go out of the house	6 (11.3)
		Take a bath	4 (7.5)
		Go to public baths or pools	3 (5.7)
		Go to hairdresser	3 (5.7)
		Sporting activities	2 (3.8)
		Walk	2 (3.8)
		Make the bed	2 (3.8)
		Sunbath on beach	1 (1.9)
		Buy clothes	1 (1.9)
		Go on a business trip	1 (1.9)
		Take a bus or taxi	1 (1.9)
		Do housework	1 (1.9)
Psychological impact/mental health	19 (86.4%)	Feel anxious	11 (21.6)
		Feel inferior	9 (17.6)
		Feel depressed	8 (15.7)
		Feel irritable	7 (13.7)
		Feel lack of hope	6 (11.8)
		Feel embarrassed	3 (5.9)
		Feel suicidal	3 (5.9)
		Perceived stigma	1 (2.0)
		Feel short tempered	1 (2.0)
		Feel dependent	1 (2.0)
		Feel the body is unclean	1 (2.0)
Social impact	19 (86.4%)	Worry about the thoughts of others	10 (21.7)
		Sexual behavior	9 (19.6)
		Make friends and meet people	7 (15.2)
		Relationships, marriages, childbearing being affected	7 (15.2)
		Worry about the reactions of others	4 (8.7)
		Social contacts and activities	4 (8.7)
		Work and career	3 (6.5)
		Feel lack of understandings of others	1 (2.2)
		Relationships with family	1 (2.2)
Employment and finances	13 (59.1%)	Reduced ability to work	6 (28.6)
		Lower income	10 (45.5)
		Cannot go to school	3 (14.3)
		Choices of a job being affected	2 (9.5)

### Symptoms, symptoms management and pain

All participants said that they had symptoms of itching and discomfort in many parts of the body. Most participants had symptoms of redness, stinging or burning, pain, and scaling (flaky skin). These symptoms were found to adversely affect their vitality (energy, vigor, and absence of fatigue) and sleep and rest (sleeping, sitting, and napping during the day), thereby affecting physical functioning. For example, a participant with psoriasis reported that he suffered from itching, which affected his sleep.

Even if I’m sleepy at night, I can’t fall asleep because it’s too itchy. Sometimes I wake up in the middle of the night. It’s so uncomfortable and I don’t know how to make it better. (Male 1, 28 years)

It hurts when I do farm work but I have to suck it up. (Male 10, 55 years)

Pain was found to be a major factor affecting patients’ quality of life, several patients stated that they would choose to be tolerant with the symptom, instead of taking painkillers to relieve their symptoms.

The symptoms of psoriasis are not itching but pain. It hurts when I bend my waist and legs. It hurts when I do other activities. But I haven't taken painkillers. I endure the pain and try to reduce my daily activities. (Male 6, 58 years)

This disease (psoriasis) is very painful, especially in the evening. It hurts and itches. I dare not take painkillers. Painkillers only make me comfortable for a while, and then it will hurt again. I think taking painkillers for a long time must be bad for my health (Female 21, 35 years)

Sometimes when the itching was very severe and participants cannot help scratching, which can lead to skin sweating, bleeding, etc.

When it itches, I’d scratch until it bleeds. In winter it would crack and hurt a lot. (Male 1, 28 years)

(It is) extremely itchy and always has flakes falling off. In the worst time, the fallen flakes from my legs would add up to 50 g. A dreadful scene. (Male 13, 62 years)

### Functioning and ADLs

Uncomfortable symptoms of psoriasis often cause participants great difficulty in various aspects of their daily life. For example, the physical symptoms of psoriatic skin lesions can significantly affect mobility including walking, carrying and climbing stairs.

The skin would grow so thick and bending down or bending legs would hurt. Moving would hurt too. (Male 6, 58 years)

I used to like going hiking in the mountains with friends. And now walking becomes a trouble for me. (Male 14, 59 years)

Psoriasis symptoms also affected participants’ specific daily activities, including clothing (which must be changed or washed more frequently) and bathing (which must be done more often).

I have to change clothes and sheets every day because of so many skin flakes. (Female 15, 30 years)

I usually have one shower each day but since I have this disease, I wish I could shower in the morning and the evening. Shower makes me feel better. (Female 8, 31 years)

Symptoms of psoriasis can have a great impact on farmers in particular. A farmer participant said that his work was mainly physical, which required a high level of physical function. He needed to walk all the time and stoop down frequently.

It hurts when I do farm work but I have to suck it up. A countryman has to work in the field. Lying around and doing nothing is never an option. (Male 10, 55 years)

### Psychological impact

Participants reported that psoriasis had a significant impact on their mental health, which can lead to an increased risk of developing mental disorders, particularly depression and anxiety. The chronic and recurring nature of this disease often brought them a feeling of hopelessness. Participants reported that they were constantly concerned with the disease and its interference with their daily life, due to unexpected outbreaks of symptoms. This concern can possibly be intensified due to their lack of control over the disease.

I don’t think the treatment is working well. I don’t hold up my hopes anymore. (Male 18, 51 years)

Sometimes I do feel pessimistic. I can’t see hope. No matter how much it costs, it will be worth it if the disease is under control and I don’t suffer this much. But after so many years and so many doctors, it’s still not cured. (Male 11, 46 years)

The disease is frustrating. I would rather have cancer. If I had cancer, people would feel sorry for me. But no one feels sorry for me with this disease. I even feel disgusted looking at myself. It looks dirty. (Male 6, 58 years)

Participants with psoriasis expressed anxiety that their relatives would be infected by them, and they often worried about their children’s health.

The only thing that scares me is the heredity of this disease. We were going to have another child, but now we’re afraid to pass this on to our kid. Sometimes my daughter says “mom, I’ve got something here on my leg. It itches.” And I would be so anxious and take her to the hospital. I’d only be relieved if the doctor says ok. (Female 21, 35 years)

Participants reported other negative feelings, including loneliness, depression and sadness, after suffering from psoriasis. A large part of the participants said that discrimination and humiliation in social interaction are the main reasons for these emotions.

Public bathhouses put up signs to forbid people with contagious diseases from going in. One time I took off my clothes to go inside. Someone asked me what the disease was. I explained but they seemed reluctant to let me in. I was so embarrassed and have never been to public bath houses ever again. (Female 17, 32 years)

I used to be confident no matter with my skin or other factors. Suddenly I got this disease. Sometimes when I’m with friends, they’d say “your skin was so good and suddenly you get this rash all over. I would be disgusted if I were you.” And I’d suddenly feel hopeless for this world and this society. (Female 8, 31 years)

I always sit in the last row on a bus because people would give you a weird look. They would stare especially in summer. (Male 1, 28 years)

### Social impact

A few participants stopped going to public places and meeting friends because of their disease, and most of the participants worried about the thoughts and reactions of others towards their disease. They reported that they felt humiliated when they exposed their bodies in public places, such as eating with others, hot springs, business trips and other social activities, without providing appropriate privacy.

I don’t go to friends’ gatherings anymore. Sometimes it shows even on my face and I feel embarrassed to go out. If I go have lunch with my friends, and the skin flakes keep falling off, I couldn’t be at ease. Even if there were no flakes, there would be white patches on the skin. I fear it would disgust others. (Female 17, 32 years)

I wouldn’t dare to share a room with others on business trips. I’m afraid if colleagues see me, they would despise me. (Male 6, 58 years)

One time a friend invited me to a hot spring bath, and I declined. I feared people might find me with the disease. (Male 11, 46 years)

I rarely go to public bathhouses in my workplace back in the day. I felt I was a dangerous beast and would scare people away. (Male 20, 70 years)

Many of the participants often felt the need to hide their disease which severely affected their self-confidence.

I’m reluctant to show my arms in my uniform at work. Always wearing long-sleeves to avoid being seen. If people ask, I feel embarrassed and upset. Sometimes it’s in the middle of an interesting conversation, and the moment would be gone. (Female 16, 46 years)

Psoriasis was also found to harm the desire for physical intimacy, as thirty percent of participants reported that psoriasis interfered with their sexual relations.

If a couple is about to have sex, it would kill the mood once the clothes are off. (Male 3, 37 years)

I have my own bed with sheets and blankets of my own. I isolate myself from my family. There’s no other way. (Male 5, 42 years)

I don’t sleep on the same bed with my wife. I sleep in another room. I loathe myself. (Male 18, 51 years)

(I’m) Not confident. For example, when looking for a marriage partner, I feel reluctant to tell the truth but it’s worse to hide it as the disease is genetic. I don’t want to spoil others’ chances. (Male 22, 31 years)

Participants also reported that their disease negatively affects courtship activities.

I know a girl patient of 25 years old. She doesn’t even want to have a relationship, fearing being despised. She always says “who would want me in this disease?” She’s worried if she gets married and the disease relapses, she’d end up in a divorce. (Female 19, 45 years)

### Negative impact on employment and finances

Psoriasis not only brought a direct financial burden to participants, but also caused serious occupational barriers. Some participants reported that psoriasis reduced their ability to work and negatively affected their income. Participants generally reported that the disease affected productivity, then the chance of employability, the choice of job and occupation, as well as the resulting income. There seemed to be an opposite relationship between psoriasis severity, works and income.

It affects work efficiency. Let’s say if I’m working on something and touched my hair accidentally. Dandruff would fall a lot and I would feel dirty and itchy and couldn’t resist scratching. If it bleeds, my concentration on work would be directly lowered. (Male 2, 30 years)

Some participants said that they were even not able to work or go to school normally.

I couldn’t work. When it’s getting serious, I have to take some rest. (Male 5, 42 years)

I think the disease is a heavy blow on people’s enthusiasm. The Spring Festival is an important time for businesses. But when the disease is back, I have to stop everything. (Female 12, 50 years)

I don’t want to go to school. Not in the mood. (Male 7, 16 years)

## Discussion

To the best of our knowledge, this is the first in-depth qualitative study in China to investigate the impact of psoriasis on QoL. Previous quantitative studies or mixed-method studies have reported that QoL is impaired in patients with psoriasis [25, 30, 31]. This was similar to what was found in our study. We found that living with psoriasis may affect patients’ physical, psychological, and social functioning, and work [22, 32–36].

In our study, patients reported symptoms including physical appearance (such as hairstyle or style of clothes), itching, redness, stinging or burning, pain, and scaling (flaky skin). This is in accordance with previous findings. For example, in a survey of 104 patients, pruritus was rated to be the worst outcome of psoriasis for over 30% of patients [37]. Chinese patients seem to have misunderstandings on painkillers [38], since they tend to treat pain relievers as drugs that can cause harm to their health or worry that using painkillers will make them addicted. Despite their life being largely affected by the symptom of pain, patients stated that they would choose to be tolerant with pain, instead of taking painkillers to relieve their symptoms.

The majority of the participants in our study reported a reduction in one or more physical activities, including outdoor activities, going to the hairdresser, choosing and buying clothes. This is equally comparable to those determined in previous quantitative studies [36, 39, 40]. We found that feeling fatigue was a prevalent physical symptom among patients, which is also similar to the findings in Skoie et al. [41], who reported that fatigue was associated with poorer quality of life. The effect of psoriasis on patients’ sexual health was found to be significant in a previous study [42]. In our study, we also found that sexual behavior was affected by psoriasis, since the affected patients and their partners tend to sleep in different rooms respectively. The disease also raises a concern regarding meeting a future partner and having children due to the lack of self-confidence caused by the disease.

Psychological disturbances were also found to be associated with psoriasis patients. Feeling depressed and anxious were the most frequently encountered psychological items in our study. Similarly, high rates of depression were reported in patients with psoriasis in previous studies [43–46]. Our study found that patients may stop going to public places and meeting friends because of psoriasis and many of them may worry about the thoughts and reactions of other people. This revealed that psoriatic patients can experience high levels of stigma, which may be due to psoriasis being a visible disease. The visibility of psoriatic lesions means that social stigmatization and rejection can be common experiences for these patients. While the public is often not well educated about psoriasis and may think the disease is contagious, individuals tend to avoid having contact with psoriasis patients, which can be agonizing for the patients and lead them to develop a distorted image of themselves [1, 42, 47, 48]. Public attitudes, if repeated, may cause anger, shame, or despair for the patients and ultimately, may make them afraid of encountering others and avoid social activities [49]. Therefore, dermatologists should consider not only the physical health but also the psychological health of their patients, who may require multidisciplinary management alongside psychologists and psychiatrists [44].

This study also found that the current social support of Chinese psoriasis patients seems to be little, as discrimination by other people was frequently mentioned in our study. This shows the lack of sufficient attention on psoriasis patients in China and the lack of publicity of psoriasis medical knowledge [50]. According to our study, visibility of skin lesions and psoriatic joint involvement may affect patients’ work productivity, career prospects and educational performance. Some studies [51, 52] found that visibility of skin lesions and psoriatic joint involvement would be more crucial in the younger patient attempting to enter the job market or finding a long-term partner. Similarly, in a study carried out in the UK, 59% of the participants expressed that psoriasis had a negative effect on their working life [36]. A Brazilian study also suggested that patients with psoriasis were likely to have an economic burden [53], because this disease requires life-long treatment which may discourage patients from working.

Some limitations of the current study should be mentioned. First, the short duration of the interviews for some inactive patients may suggest the sensitive nature of our research topic can impede the in-depth investigation. Although some patients agreed to take part in the interviews, they seemed to be reluctant to talk in-depth about their disease experience. Second, the current qualitative study is related primarily to our study setting and should be tested in further studies for wider implications.

## Conclusions

This study is the first qualitative study to report on QoL in Chinese patients with moderate-to-severe psoriasis. Results of this study showed that psoriasis has negative effects on various aspects of patients’ lives and leads to a decreased QoL. Our study detailed the effects of psoriasis on patients’ symptoms, symptoms management and pain, functioning and activities of daily living (ADLs), psychological impact, social impact, employment and finances. These data can provide a reference for studying the QoL in patients with psoriasis. It is recommended that dermatologists should consider the physical and mental health of patients, understand the plight of psoriasis patients, and provide appropriate follow-up.

## Data Availability

The data supporting the conclusion of this article are included within the article. Any queries regarding these data may be directed to the corresponding author.
